# The role of C-terminal helix in the conformational transition of an arginine binding protein

**DOI:** 10.1016/j.yjsbx.2022.100071

**Published:** 2022-08-10

**Authors:** Vinothini Santhakumar, Nahren Manuel Mascarenhas

**Affiliations:** Department of Chemistry, Sacred Heart College (Affiliated to Thiruvalluvar University, Vellore), Tirupattur District, 635601 Tamilnadu, India

**Keywords:** CTH, C-terminal helix, SBM, structure-based model, TmArgBP, thermotoga maritima arginine binding protein, Arginine binding protein, Conformational transition, C-terminal helix, Structure-based model, MD simulation

## Abstract

The *thermotoga maritima* arginine binding protein (TmArgBP) is a periplasmic binding protein that has a short helix at the C-terminal end (CTH), which is swapped between the two chains. We apply a coarse-grained structure-based model (SBM) and all-atom MD simulation on this protein to understand the mechanism and the role of CTH in the conformational transition. When the results of SBM simulations of TmArgBP in the presence and absence of CTH are compared, we find that CTH is strategically located at the back of the binding pocket restraining the open-state conformation thereby disengaging access to the closed-state. We also ran all-atom MD simulations of open-state TmArgBP with and without CTH and discovered that in the absence of CTH the protein could reach the closed-state within 250 ns, while in its presence, the protein remained predominantly in its open-state conformation. In the simulation started from unliganded closed-state conformation without CTH, the protein exhibited multiple transitions between the two states, suggesting CTH as an essential structural element to stabilize the open-state conformation. In another simulation that began with an unliganded closed-state conformation with CTH, the protein was able to access the open-state. In this simulation the CTH was observed to reorient itself to interact with the protein emphasizing its role in assisting the conformational change. Based on our findings, we believe that CTH not only acts as a structural element that constraints the protein in its open-state but it may also guide the protein back to its open-state conformation upon ligand unbinding.

## Highlights

• Probe the role of C-ter. helix (CTH) in conformational transition of TmArgBP.
• Presence of CTH almost doubles the barrier to access the closed-state.
• In the absence of CTH, the protein can fluctuate between the two conformations.
• CTH not only constraints the open-state conformation but also guides in accessing it.

## Introduction

Proteins are dynamic molecules that undergo conformational changes upon association with their respective binding partners (Bahar et al., 2010, Guo and Zhou, 2016). Upon ligand binding many proteins exhibit a conformational change from an open-state to a closed-state (Ha and Loh, 2012). Proteins exhibiting huge conformational changes are at the heart developing biosensors, where the conformational change triggered by ligand binding acts as a switch to transmit the signal (Stratton and Loh, 2011). Understanding the mechanism of such conformational changes provide vital clues about the structural modules and residues that are critical for the conformational change enabling a better insight to harness their use as biosensors as well as to develop potential inhibitors for pharmacological applications.

The protein of interest here belongs to a class of periplasmic binding proteins (PBPs), which are solute carrier proteins that bind their substrate in the periplasmic region and deliver it to their respective membrane transporters for subsequent entry into the cell (Dwyer and Hellinga, 2004, Ford and Beis, 2019, Rees et al., 2009). Many PBPs are known to exist in bacteria assisting in the transport of many diverse molecules such as amino acids, sugars, phosphate, sulphate, organic molecules and ions (Ames, 1986, Dwyer and Hellinga, 2004). In PBPs, the conformational change that occur upon ligand binding enable them to bind to their respective transporters. Once bound, the PBPs deliver their ligand for subsequent transport and in the process undergo a conformational change to their ligand-free open-state which weakens their binding facilitating their release from the transporter (Oldham et al., 2007). Thermotoga maritima arginine binding protein (TmArgBP) is a member of PBP superfamily that has a very high ligand-binding specificity for arginine (Luchansky et al., 2009, Scirè et al., 2010). TmArgBP is also a clinically valuable candidate that could be used as a biosensor to detect arginine in body fluids like blood and urine (Ausili et al., 2013). Arginine sensing in blood has a crucial role since, argininemia is an inherited disease characterized by the accumulation of arginine and ammonia in blood (Jain-Ghai et al., 2011). Hence, understanding the conformational change in TmArgBP is of great importance.

Due to a large amount of structural data available for PBPs they have been an ideal system to understand the protein dynamics and ligand induced conformational changes applying MD simulations (Bucher et al., 2011, Kienlein and Zacharias, 2020, Silva et al., 2011, Siuda and Thøgersen, 2013). Studying large-scale conformational changes using conventional all-atom MD simulations are quite challenging as they occur at time scales usually in the microsecond regime that are computationally demanding (Skjærven et al., 2011). Also, the different conformational states that are functionally relevant are separated by huge energy barriers that MD simulation cannot surpass to sculpt the underlying conformational landscape (Orellana, 2019). Coarse-grained (CG) models provide an alternate strategy to study such systems. In a CG approach the complete atomistic description of the system is replaced by mapping multiple atoms to a single bead (coarse-grain) (Kmiecik et al., 2016). This reduces the complexity of the system considerably allowing access to timescales to observe conformational changes. An earlier study had used the coarse-grained united residue (UNRES) force field for understanding the conformational transition in TmArgBP (Lipska et al., 2015). All-atom MD simulations have also been used to supplement results obtained from biophysical experiments of TmArgBP (Balasco et al., 2019, Smaldone et al., 2020).

**Fig. 1:**
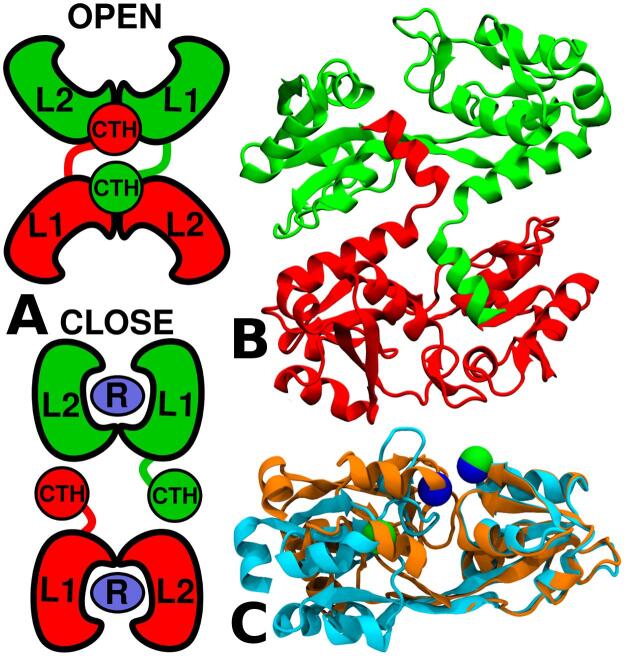
A. A cartoon representation of the domain-swapped open-state and closed-state conformation of TmArgBP. The protein consists of two lobes, L1 and L2, with arginine (ligand) binding at their interface. The two protein chains are colored in green and red. In the open-state, the C-terminal helix (CTH) docks itself at the back of the binding pocket, while in the closed-state conformation the CTH looses many of its interactions with the neighboring protein chain. B. Crystal structure of apo TmArgBP in the open-state highlighting the swapping of CTH (PDB: 4PRS). The two chains of the protein are colored in green and red. Note how the short helix at the C-terminal end (CTH) projects onto the neighbouring chain. C. Comparison of the structures of open- and closed-state of TmArgBP^ΔCTH^ colored in cyan and orange, respectively. Here the two structures are aligned with respect to the L2 domain to demonstrate the huge variation (RMSD ∼ 0.58 nm) between the open- and closed-states. The spheres pertains to Cα atoms of residues 56 and 143 in open- (green) and closed-state (blue). The distance between the Cα atoms of 56–143 is used as the reaction coordinate to monitor the conformational change.

TmArgBP crystallizes as a domain-swapped dimer in both the open-state and closed-state conformations (PDB: 4PRS (os-TmArgBP) and 4PSH (cs-TmArgBP), respectively) (Fig. 1) (Ruggiero et al., 2014). The overall fold of TmArgBP consists of a single polypeptide chain folded into two distinct lobes, called lobe 1 (L1) and lobe 2 (L2). Each of these lobes consists of a β-sheet core formed by 5 strands, surrounded by helices. The ligand binding region is located at the interface of L1 and L2. Residues 1(ILE)-90(PRO) and 190(GLY)-211(LEU) constitute L1 that contains both the *N*-terminal and C-terminal ends, while L2 consist of residues 96(GLN)-183(VAL). The two lobes are connected by two hinge segments 91(TYR)-95(GLY) and 184(LEU)-189(TYR). Finally, a short helix at the C-terminal end consisting of residues 217(ASP)-225(SER), which we refer to as the C-terminal helix (CTH), is linked through a loop, 212(LYS)-216(TYR). The swapping of CTH between the two chains is the unique feature of this protein which is not observed in other PBPs (Fig. 1). CTH has been proposed to impart stability to the protein and thought to play an important role in the formation of higher order oligomers (Ruggiero et al., 2014). The crystal structure of CTH truncated TmArgBP (TmArgBP^ΔCTH^) has also been solved in two distinct conformations, the ligand-free open-state (PDB: 6GGP) and ligand-bound closed-state (PDB: 6GGV) (Fig. 1C) (Smaldone et al., 2018). Recently, a monomeric version of the arginine-bound closed-state conformation of TmArgBP has been solved. In this structure more flexibility is introduced in the loop prior to CTH by mutating PRO-215 with GLY(2 1 5)-LYS(2 1 6) enabling the CTH to fold against the same chain (Smaldone et al., 2019).

TmArgBP undergoes a huge conformational change from open-state to closed-state with a RMSD of ∼ 0.58 nm. Interestingly, in the domain-swapped state CTH adopts two different orientations between the ligand-bound closed-state and the unliganded open-state. Only in the open-state conformation CTH docks between L1 and L2 exhibiting extensive interactions at the back of the binding pocket (Fig. 1A, B). This difference in orientation of CTH in the two conformational states intrigued us and we wanted to check if it plays a signifcant role in conformational change of the protein as well. Since CTH exhibits extensive interactions with the protein (particularly the L2 domain) only in the open-state, we wanted to check if this mechanism is in operation to restrain the open-state conformation of TmArgBP. Also, the utility of TmArgBP as a biosensor for detecting arginine is limited by the domain-swapped dimeric structure due to the possibility of formation of higher order oligomers in its recombinant form (Smaldone et al., 2016, Whitfield et al., 2015). Hence there is a huge interest to generate a monomeric version of the protein and to understand the structural elements that dictate the conformational transition. A CTH truncated version of the protein turned out to be a simple strategy to generate the monomer (Smaldone et al., 2018). Biophysical investigations suggested this truncated version to be a better scaffold for arginine sensing while imposing a destabilization effect on the ligand-bound closed-state. In another attempt to generate the monomer a single proline residue prior to CTH was mutated and the crystal structure of this mutant was solved in a closed-state conformation bound to arginine (Smaldone et al., 2019).

Experimental evidence confirms that CTH plays a crucial role in the stability of TmArgBP (Ruggiero et al., 2014). Previous study has also shown that in spite of eliminating CTH (TmArgBP^ΔCTH^) the protein retains both its stability and affinity for arginine (Smaldone et al., 2018). But as CTH adopts two different orientations between the open-state and closed-state conformations, we asked if CTH has any significant role in modulating the conformational change of TmArgBP. To this end we have modelled the monomer structure of TmArgBP in both open- and closed-states (with and without CTH, i.e TmArgBP^CTH^ and TmArgBP^ΔCTH^, respectively) to employ them in coarse-grained structure-based model (SBM) and fully solvated all-atom MD simulation. Results from SBM simulation suggests that increasing the specific interactions of CTH with the rest of the protein also increases the barrier to access the closed-state. Further, all-atom MD simulations of TmArgBP both in the presence and absence of CTH in both open- and closed-state were performed to evaluate the role of CTH. Based on our MD studies it is proposed that CTH imposes structural restraints that stabilizes the open-state thereby restricting access to the closed-state and that its absence imparts more flexibility to the protein to access both the states. Our results also indicate that CTH might play a significant role in guiding the closed-state conformation to access the open-state conformation.

## Methods

### Structures used for simulation

The structure of open- and close-state conformation of TmArgBP with CTH (os-TmArgBP^CTH^ and cs-TmArgBP^CTH^) exist as a domain-swapped dimer in which the CTH is swapped between the two protein chains. Hence, to model the monomeric version of os-TmArgBP^CTH^ we used the structural alignment tool of VMD (Humphrey et al., 1996). We took the structure of open-state TmArgBP (PDB ID: 6GGP, referred as the os-TmArgBP^ΔCTH^) and structurally aligned it with chain A of open-state TmArgBP (PDB ID: 4PRS), in which the swapped CTH segment docked at the back of the binding pocket is from chain B. Hence to create a single chain version of open-state TmArgBP with CTH, the CTH segment of chain B (residues 212–225) was linked to the terminal residue of os-TmArgBP^ΔCTH^ to generate monomeric TmArgBP with CTH, which we refer to as os-TmArgBP^CTH^. The structure of cs-TmArgBP^ΔCTH^ refers to the structure of closed-state conformation of TmArgBP without the CTH (PDB: 6GGV). Recently, the crystal structure of a monomeric version of the protein in the closed-state conformation complexed with arginine has also been solved (PDB: 6SVF, which is referred as cs-TmArgBP^CTH^) (Smaldone et al., 2019). In this structure a proline residue occurring prior to CTH is mutated (P215GK). This structure was used in our simulation to check how CTH impacts the conformational transition from closed-state to open-state in the absence of the ligand.

### SBM simulation

MD simulations of coarse-grained models offer an alternative method to study events that occur at large timescales. Several flavors of such models exist in the literature that differ in their complexity and the treatment in the conversion of all-atom representations to coarse-grained beads (Kmiecik et al., 2016, Noid, 2013, Singh and Li, 2019). One of the biggest advantage of such models is that they are easy to implement and importantly, computationally less demanding. In our study we have used a coarse-grained structure-based model (SBM) which is based on the energy landscape theory that assumes native structure as minimally frustrated (Onuchic et al., 1997). In SBMs, the parameters that make up the potential energy are constructed entirely from the native structure of the protein considering it as the global minima (Clementi et al., 2000). MD simulations of such models enable extensive sampling of events one wishes to study and have been successful to understand both folding and conformational transition in proteins (Giri Rao et al., 2016, Karanicolas and Brooks, 2002, Mascarenhas and Gosavi, 2017, Okazaki et al., 2006, Ramírez-Sarmiento et al., 2015, Whitford et al., 2007). In this work we have used a coarse-grained version of SBM where each residue is represented by a single bead located at the position of Cα (hence called the Cα-SBM) (Clementi et al., 2000, Noel et al., 2016). The potential energy (E) of the Cα-SBM used in the study can be represented as:

$$\begin{array}{c} E=\sum_{\mathrm{bonds}}K_{r}{\left(r-r_{0}\right)}^{2}+\sum_{\mathrm{angles}}K_{\theta }{\left(\theta -\theta_{0}\right)}^{2}\\ +\sum_{\mathrm{dihedrals}}^{n=1,3}K_{\varphi }\left(1-cos\left(n\left(\varphi -\varphi_{0}\right)\right)\right)+U_{\mathrm{DG}}^{\mathrm{common}}\\ +\sum_{specificcontacts\left(OS\right)}^{\;}\varepsilon_{OS\left(i,j\right)}\left[5{\left(\sigma_{\mathrm{ij}}^{\mathrm{OS}}/r_{\mathrm{ij}}\right)}^{12}-6{\left(\sigma_{\mathrm{ij}}^{\mathrm{OS}}/r_{\mathrm{ij}}\right)}^{10}\right]\\ +\sum_{specificcontacts\left(CS\right)}^{\;}\varepsilon_{CS\left(i,j\right)}\left[5{\left(\sigma_{\mathrm{ij}}^{\mathrm{CS}}/r_{\mathrm{ij}}\right)}^{12}-6{\left(\sigma_{\mathrm{ij}}^{\mathrm{CS}}/r_{\mathrm{ij}}\right)}^{10}\right]\\ +\sum_{\mathrm{non}-contacts}\varepsilon_{4\left(i,j\right)}{\left(\sigma_{\mathrm{ij}}^{\mathrm{NC}}/r_{\mathrm{ij}}\right)}^{12}\\ where,\\ U_{\mathrm{DG}}^{\mathrm{common}}=\left(1+{\left(\frac{\sigma_{\mathrm{ij}}^{\mathrm{NC}}}{r_{\mathrm{ij}}}\right)}^{12}\right)\left(1+G\left(r_{\mathrm{ij}},r_{1}^{\mathrm{ij}}\right)\right)\left(1+G\left(r_{\mathrm{ij}},r_{2}^{\mathrm{ij}}\right)\right)-1\\ where\\ G\left(r_{\mathrm{ij}},r_{n=1,2}^{\mathrm{ij}}\right)=-exp\left[-\left(\frac{{\left(r_{\mathrm{ij}}-r_{n}^{\mathrm{ij}}\right)}^{2}}{2\sigma_{n}^{2}}\right)\right] \end{array}$$

where r_0_, θ_0_ and φ_0_ refer to the native values of bonds, angles and dihedrals between 2, 3 and 4 continuous beads (Cα-atoms) with the following values for force constant, K_r_ = 100, K_θ_ = 20, K_φ(n=1)_ = 1 and K_φ(n=3)_ = 0.5, respectively. These set of value ensures that bonds, angles and dihedrals are maintained close to their native value with a weaker K_φ_ ensuring more flexibility for the dihedrals. The non-bonded interactions in SBMs were defined based on contacts, which are defined between residues that are spatially closer in the native structure of the protein and in this study these contacts were evaluated using CSU (contacts of structural units) (Sobolev et al., 1999). The contacts identified from the two conformations were divided as common contacts and state-specific (open/closed-state) contacts. Common contacts are between those residues which exhibit contacts in both the conformational states and were modeled using a dual-Gaussian (DG) contact potential ($U_{DG}^{\mathrm{common}}$), which defines two minima, one for each of the states (Lammert et al., 2009). In a typical Lennard-Jones (LJ) potential, the minima has a strong repulsive part at distances lower than the distance at which contacts are defined. Hence, if there exist contacts, such that their distance in the closed-state are shorter than the distance in the open-state (at which contact is defined), then this repulsive part of the LJ potential would act as a huge barrier to access the closed-state. Thus, modeling such common contacts as dual-Gaussian potentials helps to overcome this issue.

The parameter ε is the basic unit scale of the simulation which is set to 1 kJ/mol. In the above equation ε_OS_ and ε_CS_ set the strength of native contacts specific to the open-state and closed-state, respectively. We use the notation ε_OS_^ΔCTH^ (ε_CS_^ΔCTH^) and ε_OS_^CTH^ (ε_CS_^CTH^) to indicate the strength of open-state (_OS_) (closed-state, (_CS_)) specific contacts in the absence of CTH (^ΔCTH^) and in the presence of CTH (^CTH^), respectively. In our study, the value of ε_OS_^ΔCTH/CTH^ was fixed, while that of ε_CS_^(ΔCTH/CTH)^ was varied to the point where multiple transitions (at least 50) occurred between the two states such that both states have an equal population.^.^ The last term in the potential acts as an excluded volume component defined with σ_ij_^NC^ = 0.4 nm (which is roughly the distance between two Cα-Cα atoms) with ε_4_ = ε. In the dual-Gaussian contact potential ($U_{\mathrm{dual}-Gaussian}^{\mathrm{common}}$), r_1_^ij^ and r_2_^ij^ refers to the distance at which contact between two residues i and j are defined in the open (n = 1) and closed (n = 2) states. σ_ij_^NC^ = 0.4 nm is the excluded volume distance and σ_(n=1,2)_ = 0.05, is the width of the Gaussian well. The parameter ε_DG_ controls the depth of the Gaussian well which is set to 1. The state-specific contacts were identified using a distance criteria as follows. A contact is considered as a closed-state (open-state) specific contact if its distance in the open-state (closed-state) is more than 1.5 times the distance in the closed-state (open-state).

### SBM simulations of os-TmArgBP^ΔCTH^ and os-TmArgBP^CTH^

To assess the impact of CTH on the conformational transition, MD simulations based on the structure-based model (SBM) of (i) os-TmArgBP^ΔCTH^ and (ii) os-TmArgBP^CTH^ was performed in this study. The open-state structure was used to define SBM's bonded parameters while a dual-Gaussian function was used to define the common native contacts from both the open- and closed-state structures. Contacts specific to the two states were then added to this potential, with the strength of open-state contacts (ε_OS_^ΔCTH^ or ε_OS_^CTH^ = 1.0ε) fixed while the strength of closed-state contacts (ε_CS_^ΔCTH^ or ε_CS_^CTH^) varied to effect the conformational transition. SBM simulations were carried out using GROMACS version 4.5.4 implemented with a dual-Gaussian potential (Lammert et al., 2009). The Leapfrog stochastic dynamics integrator was used with a time step of 0.0005 (in reduced units). Structures and energies obtained from the simulations were saved every 2000 steps and the simulations were performed in a canonical (NVT) ensemble. All simulations were performed at a constant temperature of T = 100 K (in reduced units where 1 K = 0.008314). Each trajectory was run for ∼ 1 × 10^9^ steps such that the open and the closed-states were almost equally populated with sufficient number of transitions.

### All-atom MD simulations

All-atom MD simulations were performed on different monomer structures of TmArgBP (i) os-TmArgBP^ΔCTH^ (ii) os-TmArgBP^CTH^ (iii) cs-TmArgBP^ΔCTH^ and (iv) cs-TmArgBP^CTH^ (i.e simulations both in the open-state (OS) and closed-state (CS) with CTH (CTH) and without CTH (ΔCTH)) in GROMACS (v5.1.4) with AMBER99SB-ILDN forcefield (Abraham et al., 2015, Lindorff-Larsen et al., 2010). The closed-state conformation was considered without the ligand arginine. All systems were solvated in a dodecahedran box with TIP3P waters keeping a minimum distance of 1 nm between the solute and each face of the box. Counter ions were added to neutralize the charge on each system. Each of the systems were then energy minimized, initially by steepest decent followed by conjugate gradient. The system was then equilibrated allowing solvents to move freely with position restraints applied on protein heavy atoms, first under NVT and then under NPT ensemble. Production runs were then initiated from these equilibrated structures. The time-step used for the simulations were 2 fs with V-rescale and Perrinello-Rahman used for maintaining temperature and pressure at the desired value of 300 K and 1 atm, respectively. All H-bonds were restrained using the LINCS algorithm. Verlet-cutoff scheme was used for treating short range non-bonded interactions using a cutoff of 1.0 nm, while the long-range Coulombic interactions were treated using PME. All systems were run under periodic boundary conditions and the coordinates from each production run were saved every 2000 steps.

## Results and discussion

TmArgBP exists as a dimer with the C-terminal helix (CTH) mutually swapped between the two chains. To study the functional implications of CTH, a single chain version of TmArgBP was constructed such that the CTH docks on to the same chain. Using this construct of the single chain protein we set out to investigate the role of CTH on the conformational transition of the protein. The results from MD simulations obtained by applying a dual-Gaussian SBM model are presented first followed by results from all-atom MD simulation. The native centric Cα-SBMs offers a distinct advantage in studying conformational transition with the assumption that the mechanism of conformational transition depends solely on the two end states and its posit lies in taking advantage of the difference in the distribution of contacts between them (Jayanthi et al., 2020, Okazaki et al., 2006, Ramírez-Sarmiento et al., 2015, Whitford et al., 2007). In this study a dual-Gaussian SBM potential is defined from the knowledge of common contacts from both the open- and closed-states of TmArgBP and the structural information unique to both the states are encoded as specific contacts. The strengths of all contacts present in the open-state conformation were maintained at a specific value (ε_OS_^(ΔCTH/CTH)^ = 1.0ε) while those of the closed-state (ε_CS_^(ΔCTH/CTH)^) were varied to enforce the conformational transition. In proteins that undergo huge changes in secondary structure upon conformational transition, incorporating contacts alone might be insufficient (Lin et al., 2014, Okazaki et al., 2006, Ramírez-Sarmiento et al., 2015). In the protein of interest here, there are no significant changes in the secondary structural elements and hence incorporating only the differences in contacts between the two conformational states was considered sufficient.

### SBM simulation of TmArgBP^ΔCTH^

**Fig. 2:**
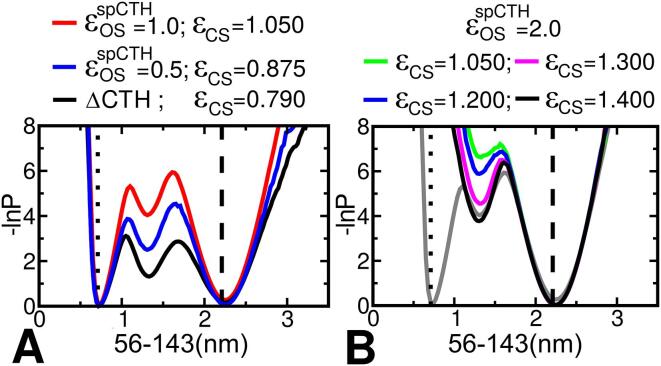
A. The free energy plots as a function of the distance between the Cα atoms of residues 56–143 for os-TmArgBP. The black curve corresponds to the simulation started with os-TmArgBP^ΔCTH^ (the open-state in the absence of CTH) with strength of open-state specific contacts fixed at ε_OS_ = 1.0. The blue and the red curves correspond to simulation started with the open-state in the presence of CTH (os-TmArgBP^CTH^) with a value of ε_OS_^spCTH^ (specific CTH contacts in the open-state) set to 0.5 and 1.0, respectively. In the simulation of os-TmArgBP^CTH^ the strength of open-state specific contacts evaluated from os-TmArgBP^ΔCTH^ are still present (ε_OS_ = 1.0). B. Simulation of os-TmArgBP^CTH^ with ε_OS_^spCTH^ = 2.0. The different curves correspond to simulation performed with increasing value of ε_CS_. The grey curve is indicated for comparison, which corresponds to the red curve in A.

The first SBM simulation was started from the open-state structure of TmArgBP in the absence of CTH (os-TmArgBP^ΔCTH^). A total of 528 contacts were identified as common contacts in os-TmArgBP^ΔCTH^ and were treated as dual Gaussians with ε_DG_ = 1.0. This means that these contacts have two minima, one defined at the distance in their open-state and the other defined at the distance in their closed-state. In os-TmArgBP^ΔCTH^ 14 contacts were identified as open-state specific contacts and in order to stabilize the starting open-state conformation these contacts were included with ε_OS_^ΔCTH^ = 1.0. In the simulation started from the os-TmArgBP^ΔCTH^ inclusion of 33 closed-state specific contacts, with ε_CS_ = 0.790, was sufficient to promote conformational transition between the two states (Fig. 2A). The distance between the Cα atoms of residues 56–143 served as the reaction coordinate to monitor the progress of the conformational change. It is evident from the figure that the distance between 56 and 143 accessed by the open-state and closed-state structures during the conformational transition are almost similar to the distances in their respective native states. Hence, our SBM simulation protocol of defining common-contacts as dual-Gaussians with state specific contacts as 10–12 LJ potential is justified.

### SBM simulation of TmArgBP^CTH^

Encouraged by the results obtained from os-TmArgBP^ΔCTH^, simulation of os-TmArgBP^CTH^ was undertaken to monitor the impact of CTH in the conformational transition. The rationale for our interest in probing the role of CTH in conformational transition of TmArgBP arises from the observation that it adopts two different orientations in the open-state and closed-state (Ruggiero et al., 2014). CTH in the open-state conformation interacts extensively with L2 by docking between the two lobes at the back of the ligand binding pocket. In the closed-state it looses many of these interactions with L2 and moves close to L1. The inclusion of open-state specific contacts arising from the presence of CTH is the main difference in this simulation. In comparison to 528 common-contacts in os-TmArgBP^ΔCTH^, 558 contacts in os-TmArgBP^CTH^ were identified as common contacts and were treated as dual Gaussians. In os-TmArgBP^ΔCTH^, 14 contacts were identified as open-state specific contacts, while in case of os-TmArgBP^CTH^, 27 contacts filtered as open-state specific contacts. The additional 13 contacts arising exclusively from the inclusion of CTH suggesting a strong interaction of CTH only in the open-state.

As CTH exhibits many contacts only in the open-state, we hypothesize that it is likely to play a role in stabilizing the open-state conformation of the protein and also that by garnering interactions with L2 it may contribute towards conformational transition from closed-state to open-state upon ligand unbinding. Hence, strengthening interactions of CTH could make the protein that much more difficult to access the closed-state, which should be reflected in the free energy profile. As a result, in subsequent SBM simulations, the strength of open-state specific contacts obtained from os-TmArgBP^ΔCTH^ (ε_OS_) were maintained at a constant value (ε_OS_ = 1.0) while the strength of open-state specific contacts involving CTH (ε_OS_^spCTH^) were gradually increased to assess how conformational transitions are related to the strengths with which CTH interacts with the protein. With this idea in mind the specific contacts of the CTH in the open-state were varied to (i) ε_OS_^spCTH^ = 0.5 (i.e at half the strength compared to the strength of rest of the open-state contacts in os-TmArgBP^CTH^, i.e ε_OS_^spCTH^ = 0.5 × ε_OS_) (ii) ε_OS_^spCTH^ = 1.0 (ε_OS_^spCTH^ = 1.0 × ε_OS_) and (iii) ε_OS_^spCTH^ = 2.0 (ε_OS_^spCTH^ = 2.0 × ε_OS_). The total number of closed-state specific contacts in os-TmArgBP^CTH^ was 35 (ε_CS_^CTH^) and we observed that the value of ε_CS_^CTH^ required to induce transition increased as we increased ε_OS_^spCTH^. The value of ε_CS_^CTH^ required to obtain sufficient transition between the two states when ε_OS_^spCTH^ was set to 0.5 and 1.0, was 0.875 and 1.050, respectively (Fig. 2B). When CTH specific contacts were included at ε_OS_^spCTH^ = 0.5 there was a considerable increase in the energy barrier (when compared to simulation of os-TmArgBP^ΔCTH^) which increased to almost twofold when ε_OS_^spCTH^ = 1.0, suggesting a direct link between the strength of interaction that CTH exhibits and the restraint it imposes on the open-state to access the closed-state.

To investigate the impact of CTH on the conformational transition, the strength of CTH specific contacts were increased further to ε_OS_^spCTH^ = 2.0 and it was observed that no transition to closed-state was possible even when the strength of closed-state specific contacts were increased significantly (ε_CS_^CTH^ = 1.400). By comparing the two SBM simulations we infer that as the strength of contacts specific to CTH were increased the strength at which closed-state contacts needs to be incorporated also increases. Based on these simulations we concluded that interactions of CTH with L2 stabilizes the open-state and that these interactions needs to be perturbed to access the closed-state. Thus we hypothesize that CTH influences the conformational transition and that one can modulate the conformational change by tuning the specific interactions of CTH in the open-state. It also appears that a very strong interaction of CTH with the rest of the protein could even potentially lock the conformation in the open-state.

### All-atom (AA) MD simulation of TmArgBP

Results of SBM simulation obtained from os-TmArgBP^ΔCTH^ and os-TmArgBP^CTH^ suggested that the presence of CTH imposes structural restraints in the open-state so as to prevent access to the closed-state. Based on this result we took up all-atom MD simulations of (i) os-TmArgBP^ΔCTH^ (ii) os-TmArgBP^CTH^ (iii) cs-TmArgBP^ΔCTH^ (iv) cs-TmArgBP^CTH^ to assess the dynamics of TmArgBP in different conformational states and also to probe the influence of CTH in these states. In order to monitor the conformational changes in these simulations we use the same reaction coordinate as in SBM, which is the distance between the Cα atoms of 56–143. We also shed light on some of the fundamental interactions that CTH exhibits when it is present in the protein. Results from MD simulation of cs-TmArgBP^CTH^ also demonstrates how CTH aids in the conformational transition from a closed-state to open-state.

### AA simulation of os-TmArgBP^ΔCTH^

**Fig. 3:**
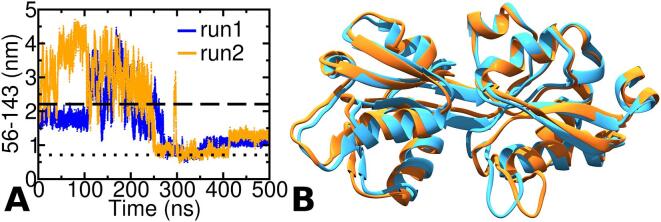
MD simulation results of os-TmArgBP^ΔCTH^. A. The distance between the Cα atom of residue 56–143 plotted as a function of time for the two independent simulations of monomeric version of os-TmArgBP^ΔCTH^. In both these simulations the distance between 56 and 143 decreases to a value that is indicative of a conformational transition to the closed-state. B. A snapshot from r1-TmArgBP^ΔCTH^ (orange) ∼ 256 ns, the snapshot with the lowest RMSD of 0.18 nm with the closed-state) compared with X-ray structure of the closed-state (cyan).

Two independent simulations of os-TmArgBP^ΔCTH^ (referred as r1-os-TmArgBP^ΔCTH^ and r2-os-TmArgBP^ΔCTH^) were first carried out. In the first simulation run (r1-os-TmArgBP^ΔCTH^) we observed the protein to be quite stable in its open-state conformation (until 100 ns) as the distance (56–143) is close to the value in the open-state (Fig. 3A). This is followed by a period of ∼ 160 ns where the protein exhibits high degree of fluctuations. At ∼ 265 ns, the protein gains access to closed-state and from there on maintains this conformation for the rest of the simulation. A second independent simulation was also carried out for os-TmArgBP^ΔCTH^ and a similar conformational switch to closed-state was observed (Fig. 3A). In the second independent run (r2-os-TmArgBP^ΔCTH^) the protein initially displayed a big change in conformation and reached the closed-state ∼ 250 ns. The closed-state conformation accessed during the simulation is in close resemblance to the experimental structure (Fig. 3B). It is also to be noted here that in the last ∼ 100 ns, there is a slight increase in the distance between 56 and 143 suggesting a penchant for conformational change.

### AA simulation of os-TmArgBP^CTH^

**Fig. 4:**
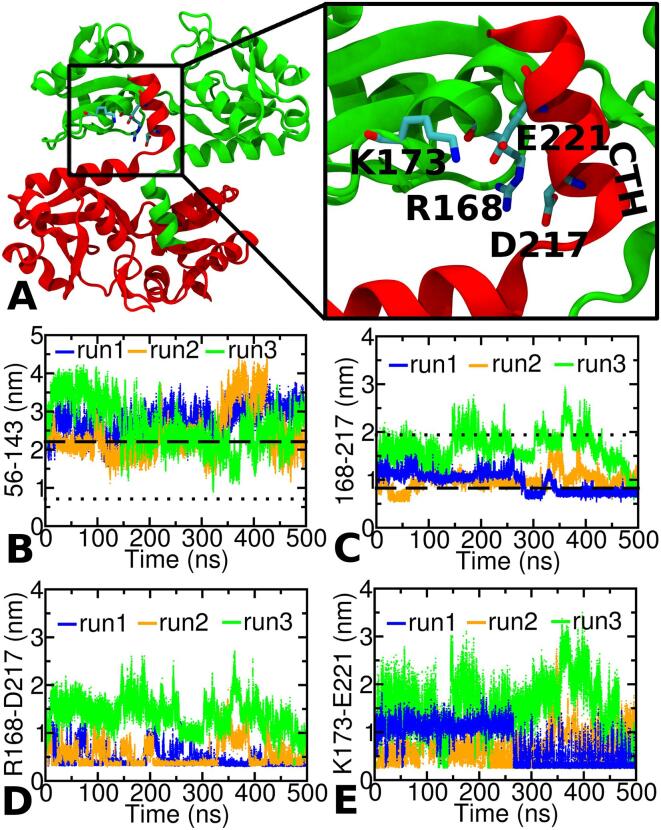
A. The overall structure of domain-swapped os-TmArgBP. The two chains are colored in green and red. The boxed region highlights the ionic interactions of CTH with its neighboring chain. The two acidic residues in CTH, namely D217 and E221, form salt-bridge like interaction with K173 and R168, respectively. Simulation results for os-TmArgBP^CTH^. The distance between the Cα atoms of residue 56–143 (B) and 168–217 (C) plotted as a function of time for three independent simulations of monomeric version of os-TmArgBP^CTH^. While the Cα distance between 56 and 143 monitors the open to close transition, that between 168 and 217 is a good proxy to monitor the closeness of CTH to the protein. To monitor the salt-bridge interactions across the three independent runs of os-TmArgBP^CTH^, the distance between the charged side-chain centers of residues R168-D217 (D) and K173-E221 (E) is plotted. The plot is the closest distance between any of the oxygen atoms of acidic residues and the nitrogen atoms of basic residues. In this study we set a cut-off of 0.5 nm for measuring the salt-bridge. Please note that in 168–217, we refer to the distance between the Cα-atoms of residues 168 and 217 while in R168-D217, we refer to the distance between the charged side-chain centers of the two residues.

To check if CTH stabilizes the open-state thereby restraining access to the closed-state, we performed three independent MD simulation runs of os-TmArgBP^CTH^. Monitoring the distance between 56 and 143 in all the three runs implies that the protein remains predominantly in its starting open-state conformation (Fig. 4B). In r3-os-TmArgBP^CTH^, the protein does gain access to the closed-state, but only for a short duration (at ∼ 320 ns) reverting immediately back to the open-state. Hence, unlike the previous simulations of r1/r2-os-TmArgBP^ΔCTH^, the protein does not remain in the closed-state for quite long (Fig. 4B). To assess the orientation of CTH, the distance between Cα atoms of 168–217 were monitored (Fig. 4C). We chose these residues as 168 lies at L2 lobe of the protein while 217 lies roughly in the middle of CTH and thus monitoring the distance between these residues is a good indication of how close is CTH to L2. In case of both r1/r2-os-TmArgBP^CTH^, the distance between the Cα-atoms of 168–217 is very close to that observed in the open-state, and the distance between 56 and 143 maintain a value indicative of an open-state conformation. On the other hand, in r3-os-TmArgBP^CTH^, we observed the protein to access the closed-state momentarily and then reverting back to the open-state conformation. Interestingly, in this simulation the distance between the Cα atoms of 168–217 is quite high compared to the previous two simulations, suggesting that the helix is far away from the L2 lobe. From these three simulations we infer that in r1- and r2-os-TmArgBP^CTH^, where the helix interacts with L2, the protein remains predominantly in the open-state but in r3-os-TmArgBP^CTH^, where the helix seems to move away from L2 (Fig. 4C), the protein has a greater inclination towards closed-state conformation, suggesting that the interaction of CTH is critical to stabilize the open-state.

### Salt-bridges that stabilize the open-state

In the domain-swapped open-state conformation of the protein (PDB: 4PRS) two acidic residues from the swapped CTH segment, D217 and E221, had salt-bridge like interactions with basic residues, R168 and K173, respectively (Fig. 4A). Based on this observation we monitored for all possible salt-bridge interactions between any residues of CTH and L2 using the salt-bridge plugin in VMD. We observed that only the above sets of residues display such ionic interactions. To monitor for salt-bridge interactions across the three independent runs of os-TmArgBP^CTH^, we plotted the distance between the charged side-chain centers of residues R168-D217 (Fig. 4D) and K173-E221 (Fig. 4E). In both r1/r2-os-TmArgBP^CTH^ the distance between the charged-centers of R168-D217 remains predominantly close to 0.5 nm, where one could expect strong ionic interactions between their side-chains. The same for K173-E221 across the three runs shows that only in r1-os-TmArgBP^CTH^, the ionic interactions begin to dominate, that too only after ∼ 250 ns. But as observed for R168-D217, the value of the distance does not remain close to 0.5 nm, but varies over a range between 0.5 and 1.0 nm. In the case of r2-os-TmArgBP^CTH^, the distance K173-E221 varies between 0.5 and 1.0 nm during the entire duration of the simulation. This suggests that in both these runs, some amount of ionic interactions does prevail between K173-E221 but are not as effective as that of R168-D217.

**Fig. 5:**
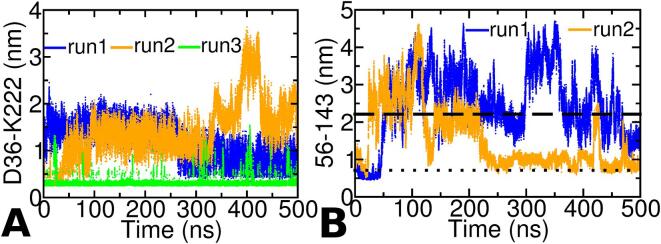
A. Distance between the charged centers of D36-K222 in r1/r2/r3-TmArgBP^CTH^ as a function of simulation time. The distance in case of r3-TmArgBP^CTH^ alone is quite stable ∼ 0.4 nm indicative of a strong salt-bridge. In both r1/r2-TmArgBP^CTH^ the distances are not as stable as that observed for r3-TmArgBP^CTH^. B. Distance between the Cα-residues of 56–143 as a function of time for the two independent simulation runs starting from the closed-state structure of TmArgBP without CTH (r1/r2-cs-TmArgBP^ΔCTH^). In both simulations, the protein escapes to the open-state within ∼ 75 ns and in r2-TmArgBP^ΔCTH^ one can see the protein reverting back and forth between the two states.

In complete contrast, the same distance between the charged centers of R168-D217 and K173-E221 in r3-os-TmArgBP^CTH^ are quite high (greater than 1 nm) for most of the simulation runtime, indicating stable ionic interactions are not formed to a significant extent. We then asked the question, why is this happening in r3-TmArgBP^CTH^ alone and not in the other two simulations. It was observed that only in case of r3-TmArgBP^CTH^ the distance between the charged centers of residue D36 (L1) and K222 (CTH) were stable through out the entire duration of the simulation at a value of ∼ 0.4 nm, indicating a strong ionic interaction between them (Fig. 5A). We believe that this ionic interaction formed early in the simulation could hinder the migration of CTH towards the L2, which is required to stabilize the open-state as in case of r1/r2-TmArgBP^CTH^.

### AA simulation of cs-TmArgBP^ΔCTH^

In the AA simulation of os-TmArgBP^ΔCTH^ the value of the distance 56–143 evaluated to monitor the conformational change goes from ∼ 2.2 nm (open-state) to ∼ 0.7 nm (closed-state) within 275 ns. This suggests that the open-state of TmArgBP in the absence of CTH (os-TmArgBP^ΔCTH^) can access the closed-state conformation without the ligand. But in the last 100 ns of the simulation both the runs (r1/r2-os-TmArgBP^ΔCTH^) show signs of a slight increase in the distance suggesting that a longer run might revert the conformation back to the open-state. To weigh this possibility simulation of cs-TmArgBP^ΔCTH^ was undertaken. Our idea was to check whether in the absence of CTH the ligand-free closed-state conformation (cs-TmArgBP^ΔCTH^) of the protein can switch to an open-state conformation. Also from our previous simulation of os-TmArgBP^CTH^, our main conclusion was that CTH acts as a structural restraint that stabilizes the open-state, hence we were interested in the simulation of cs-TmArgBP^ΔCTH^ to check whether the protein gains access to the open-state and if it does, can it remain there long enough (as there is no CTH to stabilize the open-state) or does it revert back to the closed-state.

In both the runs of cs-TmArgBP^ΔCTH^ (r1/r2-cs-TmArgBP^ΔCTH^) the protein approaches the open-state conformation within ∼ 75 ns (Fig. 5B). In case r1-cs-TmArgBP^ΔCTH^ the distance of 56–143 remain above 2.2 nm for most of the time, which is the distance observed in the native structure of the protein, suggesting the protein to exist predominantly in the open-state. In case of r2-cs-TmArgBP^ΔCTH^ one can see that the distance first increases, suggesting a transition to the open-state, but ∼ 125 ns it comes down to ∼ 1 nm, but within the next ∼ 25 ns it again reaches the open-state. Again at ∼ 425 ns one can observe the protein to access the open-state and then reverting back to closed-state. Thus, in r2-cs-TmArgBP^ΔCTH^ we observe the protein reverting back and forth between the two states. In case of r1-cs-TmArgBP^ΔCTH^ such a trend is not observed, although in the last ∼ 50 ns of the simulation, the distance between 56 and 143 is considerably reduced suggesting a tendency to access the starting closed-state conformation. Based on our data we suggests that in the absence of CTH, a closed-state conformation can also access the open-state. We also believe that the open-state accessed during the simulation is not stabilized due to the lack of CTH.

### AA simulation of cs-TmArgBP^CTH^

**Fig. 6:**
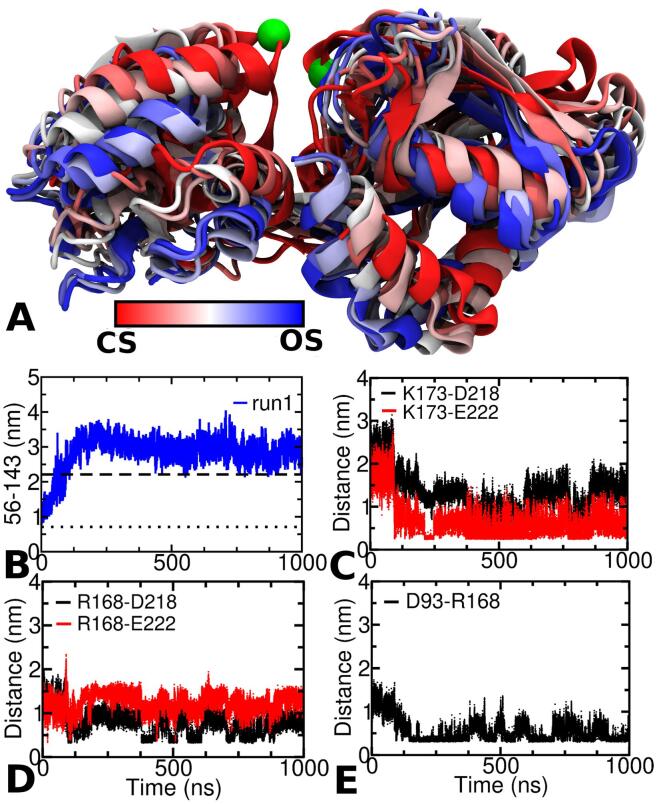
A. Initial snapshots from 0 to 125 ns at intervals of 25 ns highlighting the conformational transition from closed-state (CS) to open-state (OS). The first (CS) and last (125 ns) snapshots are coloured red and blue, respectively, with intermediate (25 ns) snapshots colored according to the color bar. In the figure movement of the CTH and its insertion between the two lobes is clearly depicted. The location of 56–143 (Cα) atoms in the closed-state is indicated as green sphere. B. The distance between the Cα atom of residues 56–143 plotted as a function of time used to monitor the conformational change. C. Distance between the charged-centers of K173 (in L2 that is oriented towards CTH) and acidic residues (D218 and E222) in CTH. D. Distance between the charged-centers of R168 (in L2 that is oriented towards CTH) and acidic residues (D218 and E222) in CTH. E. Distance between the charged-centers of R168 and D93 (at the hinge).

Recently, the co-crystal structure of cs-TmArgBP^CTH^ bound to arginine has been solved, in which the protein exist as a monomer (Smaldone et al., 2019). Hence, to study the impact of CTH on the conformational transition, we decided to run a very long simulation of this structure after eliminating the ligand. Our objective was to investigate whether in the absence of ligand the protein is able to access the open-state and also to probe the role of CTH in such a transition. From our simulation it was observed that the protein gains access to the open-state within ∼ 125 ns and it remained in this conformation till the end of the long simulation run. To check if this conformational transition was aided by CTH, snapshots at intervals of 25 ns was obtained from the initial 125 ns of the simulation (Fig. 6A). These snapshots not only highlight the overall domain motions of the protein but also show how the orientation of CTH changes during this transition. During the initial phase of the simulation there is an overall change in the conformation from open-state to closed-state. The distance between residues 56–143, which is used as the proxy to monitor the conformational change between the two states exhibits a huge difference within the ∼ 125 ns of the simulation, clearly suggesting a conformational change towards the open-state (Fig. 6B). One can also clearly see that as the two lobes move away from each other, a conformational change from closed-state to open-state occurs and simultaneously the CTH also begins to insert itself between the two lobes (Fig. 6A). From here on till the end of the simulation (1000 ns) the CTH is locked strongly between the two lobes suggesting that the open-state is stabilized by this CTH. In our previous simulation of os-TmArgBP^CTH^, the CTH domain exhibited salt-bridge interactions with residues from the L2 lobe. In this simulation it was observed that out of the two positively charged residues in L2, only K173 exhibited salt-bridge like interactions with E222 from CTH (Fig. 6C). Another positively charged residue at L2 oriented towards CTH capable of forming salt-bridge interaction was R168. Upon comparing the distances between the charged-centers of R168 with D218 and D222, we observed only R168-E222 to engage in some sort of salt-bridge interactions, while the distance between R168-E222 were too long to have any appreciable interactions (Fig. 6D). In order to investigate why R168 did not enjoy a similar ionic interaction as observed for K173, we looked for any other residue that is engaged with R168. We noticed that R168 enjoyed a stable ionic interaction with D93 from the hinge which probably prevents it from interacting strongly with L2 (Fig. 6E). The impact of CTH binding and its implication on conformational change are being probed in a detailed manner, which we will describe elsewhere.

Experimental evidence suggests that although removal of CTH (TmArgBP^ΔCTH^) retains the affinity of the protein for arginine but in comparison to TmArgBP^CTH^_,_ it completely alters the stability of the protein in ligand-free and ligand-bound forms. The authors argue that removal of CTH exposes several hydrophobic residues at the back of the binding pocket that causes destabilization of the ligand-bound state compared to that of the ligand-free state. It is proposed that in the presence of arginine the rigidity of the closed-state conformation constraints the protein so as to forbid local structural rearrangements that prevents exposure of these hydrophobic residues. Hence the presence of ligand in the truncated version of TmArgBP induces some unstablility while in the full length version of TmArgBP the ligand-bound closed-state is the most stable (Smaldone et al., 2018). Our results too corroborate with this observation. Taking together the results of the truncated version of the protein in both the conformational states (os-TmArgBP^ΔCTH^ and cs-TmArgBP^ΔCTH^), it is quite clear that neither of them is stable in their starting conformation. Results from both these simulations show that the protein undergoes significant change to even access the other conformation state. In contrast the protein is quite stable in its open-state conformation in the presence of CTH (TmArgBP^CTH^).

### Mechanism of conformational transition

Conformational changes triggered upon ligand binding are typically classified into two models, induced-fit and conformational selection (Changeux and Edelstein, 2011). In the induced-fit mechanism, the ligand binds to the open-state, which triggers the conformational change to access the closed-state. In the conformational selection scenario the protein is flexible enough to transiently gain access to the closed-state and the ligand then selects this conformation to bind. Hence, in the induced-fit mechanism the ligand is essential to unlock the closed-state conformation, while in the conformational selection it appears that the protein possesses inbuilt intrinsic flexibility to access the closed-state at least for a very short span (Jayanthi et al., 2020). In the absence of the ligand many multi-domain proteins exist in their open-state, which undergo a conformational change to the closed-state upon ligand binding and these systems provide an ideal set-up to understand conformational changes.

TmArgBP exists in the open-state conformation in the presence of CTH (os-TmArgBP^CTH^) where the CTH at the back of the binding pocket has strong interactions with L2 (Fig. 4). Upon ligand binding, CTH loses many of these interactions and its orientation is such that it no longer faces L2. In the all-atom MD simulation of os-TmArgBP^CTH^ the protein remains in the open-state while in the simulation of os-TmArgBP^ΔCTH^ the protein undergoes a conformational change to the closed-state, suggesting that CTH interacts with the protein so as to prevent access to the closed-state. This implies that CTH is an efficient structural element to constrain the protein in its open-state conformation. In the ligand-bound closed-state conformation the orientation of CTH is such that it has no interactions with the protein. In order to operate by induced-fit the ligand has to bind to the open-state conformation of the protein which favors optimal binding. Therefore, the need to stabilize the open-state to acquire the incoming ligand is essential and we strongly believe that the main role of CTH in the protein might be to stabilize the open-state to facilitate ligand binding. We believe that once ligand binds, it is capable of disrupting the interactions of CTH thereby enabling access to the closed-state, suggesting an induced-fit mechanism in TmArgBP^CTH^. Our results from the simulations of os/cs-TmArgBP^ΔCTH^ suggests that without CTH the protein is capable of accessing both the states and we hypothesize that elimination of CTH imparts intrinsic flexibility to the protein. Our simulations therefore suggests that TmArgBP^CTH^ is likely to invoke the induced-fit mechanism to bind its ligand while elimination of CTH (TmArgBP^ΔCTH^) could potentially convert this protein to follow the conformational selection mechanism.

### CTH in other related proteins

Our findings show that CTH restraints the open-state conformation, and we hypothesize that it can be used to modulate the conformational change. We then asked whether this CTH is unique to this protein or is it a feature shared by other members of the family too, so we performed a BLASTp search of TmArgBP^CTH^ restricting to structures within the PDB. We were successful in obtaining 35 structures (after removing repetitive sequences) with a helix at the C-terminus (kindly refer Supplementary Material, Table ST1). The sequence alignment of all sequences from the preceding list of 35 structures with a sequence identity value greater than 30 % strongly suggests that CTH is highly conserved (kindly refer Supplementary Material, Fig. S1). We then ran an MD simulation of ArtJ, an arginine-, lysine-, and histidine-binding protein from the Geobacillus Stearothermophilus (Vahedi-Faridi et al., 2008). The co-crystal structure of this protein bound to lysine is available in the closed-state conformation (PDB: 2PVU, referred as GsArtJ). GsArtJ was chosen as it aligned structurally well with cs-TmArgBP (with an RMSD of 0.167 nm). This MD run was intended to check if the closed state conformation could be aided by CTH to gain access to the open state conformation. Similar to what was observed for CTH in cs-TmArgBP, as the CTH of GsArtJ shifts towards L2 the protein simultaneously gains access to an open-state conformation (Fig. S2). Hence, CTH could play a vital role in controlling the conformational transition in this family of periplasmic binding protein which could be utilized to modulate the affinity for the ligand.

### Experimentally testable predictions

In many biotechnological applications it is necessary to ascertain the molecular mechanism of conformational change and the structural factors that control these events. Such an understanding of conformational change is highly informative to biochemists to decide upon structural parameters that are to be modified to increase the potency of the protein towards its binding partners, as well as to increase their specificity or make it possible for them to bind to an entirely new set of ligands (Walker et al., 2010). In the protein of interest, TmArgBP, which has high affinity towards arginine our objective was to enlighten these structural parameters such that one can take clues from our study to design suitable experiments that could potentially modulate the binding affinity of this protein.

Our SBM simulation studies comparing the conformational changes occurring in TmArgBP both in the presence and absence of CTH clearly shows that the presence of CTH constrains the open-state which eventually increases the barrier to access the closed-state conformation. The results of all-atom MD simulations corroborate with the results of SBM simulation and it indicates that the presence of CTH equips the protein to maintain its open-state conformation. Salt-bridge interactions of CTH with L2 were observed in the simulation of os-TmArgBP^CTH^, and we hypothesized that these interactions link the CTH with the protein, thereby stabilizing the open-state conformation. A CTH-like helix called the spine-helix (SH) has been observed to perform a similar role in MBP (maltose-binding protein), a structurally similar family of periplasmic binding protein (Jayanthi et al., 2020). It has been demonstrated that changing the SH residues to more bulky ones (I329W) lengthens the distance between the SH and the *N*-terminal domain at the back of the binding pocket (Telmer and Shilton, 2003). This increase in distance propels the protein's closing motion, increasing the protein's affinity for its ligand by several orders of magnitude. In our study we have shown how salt-bridges play a critical role in the binding of CTH and we believe that destabilizing the interaction of CTH with the protein could potentially destabilize the open-state thereby alleviating their access towards the closed-state with increased affinity for the ligand.

## Conclusion

TmArgBP is a periplasmic binding protein that binds arginine with high selectivity. Using both coarse-grained structure-based model (SBM) and all-atom MD simulation the conformational transition in TmArgBP have been studied and particularly the role of CTH in dictating the conformational transition have been probed. Results from dual-Gaussian SBM of os-TmArgBP^ΔCTH^ and os-TmArgBP^CTH^ suggests that inclusion of open-state specific contacts of CTH impedes access to the closed-state. We also observed that the barrier to access the closed-state increased as the strength of open-state specific interactions of CTH were increased hinting that the interaction of the CTH at the back of the binding pocket constraints the conformational transition. Multiple independent unbiased all atom MD simulations started from os-TmArgBP^ΔCTH^ accessed the closed-state conformation within ∼ 250 ns while that of os-TmArgBP^CTH^ constrained the conformation in the open-state. These findings imply that CTH interaction is such that it applies structural restraint in the open-state, preventing conformational access to the closed-state. Interestingly, simulation initiated from cs-TmArgBP^ΔCTH^ not only accessed the open-state conformation but also transitioned back and forth between the two-states, implying open-state accessed during the simulation is not stabilized. Finally, simulation started from unliganded cs-TmArgBP^CTH^ accessed the open-state during the initial phase of the simulation with CTH aiding the conformational transition. In conclusion, this study using a mutliscale MD simulation approach suggests two important roles for CTH in TmArgBP. First, CTH in the open-state interacts with the protein in a manner so as to stabilize it and second, it also aids in accessing the open-state conformation upon ligand unbinding.

## CRediT authorship contribution statement

**Vinothini Santhakumar:** Methodology, Investigation, Writing – original draft. **Nahren Manuel Mascarenhas:** Conceptualization, Funding acquisition, Investigation, Writing – review & editing.

## Declaration of Competing Interest

The authors declare that they have no known competing financial interests or personal relationships that could have appeared to influence the work reported in this paper.
