# Supplementary material for: The role of C-terminal helix in the conformational transition of an arginine binding protein
Source: J Struct Biol X. 2022 Aug 10;6:100071. doi: 10.1016/j.yjsbx.2022.100071 (PMC9402392; doi:10.1016/j.yjsbx.2022.100071)
Supplement: Supplementary data 1 [file mmc1.docx]

**Supplementary Material**

**The role of C-terminal helix in the conformational transition of an arginine binding protein**

**Vinothini Santhakumar**^1^ **and Nahren Manuel Mascarenhas**^1*^

^1^Department of Chemistry, Sacred Heart College (Affiliated to Thiruvalluvar University, Vellore) Tirupattur District 635601, Tamilnadu, INDIA

^*^Corresponding author: mailnahren@gmail.com

| **Table ST1**. List of 35 non-redundant structures (from PDB) that filter from the BLASTp search of TmArgBP^CTH^. |
| --- |
| 1GGG, 1XT8, 2IEE, 2M8C, 2O1M, 2PVU, 2Q88, 2Y7I, 2YJP, 2YLN, 3DEL, 3H7M, 3HV1, 3K4U, 3KZG, 3QAX, 3TQL, 3VV5, 4F3P, 4G4P, 4GVO, 4I62, 4OHN, 4YMX, 4Z9N, 4ZEF, 5EYF, 5HPQ, 5IKB, 5ORE, 5OT8, 5OWF, 6A80, 6DET, 6H2T |


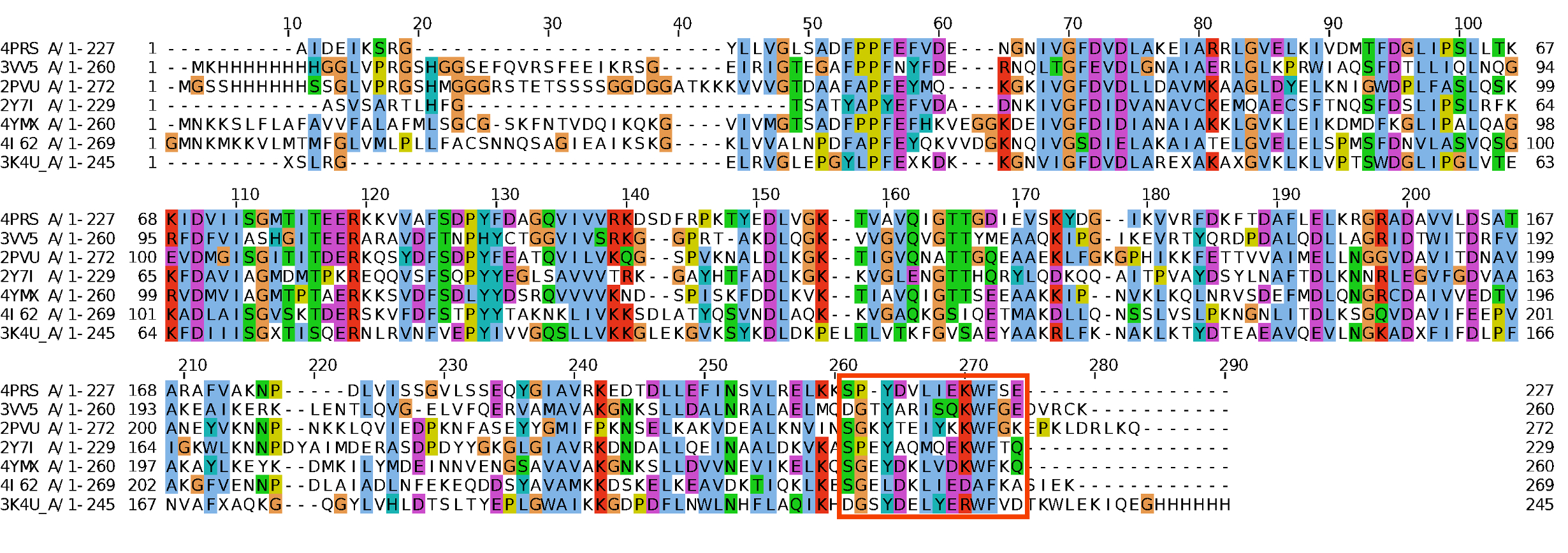
**Figure S1.** **A.** The sequence alignment of similar proteins (sequence identity >30%) obtained from ‘BLASTp’ search of TmArgBP^CTH^. The box colored in orange is the region corresponding to the CTH of TmArgBP, suggesting that CTH is conserved well among the different families of periplasmic-binding protein.


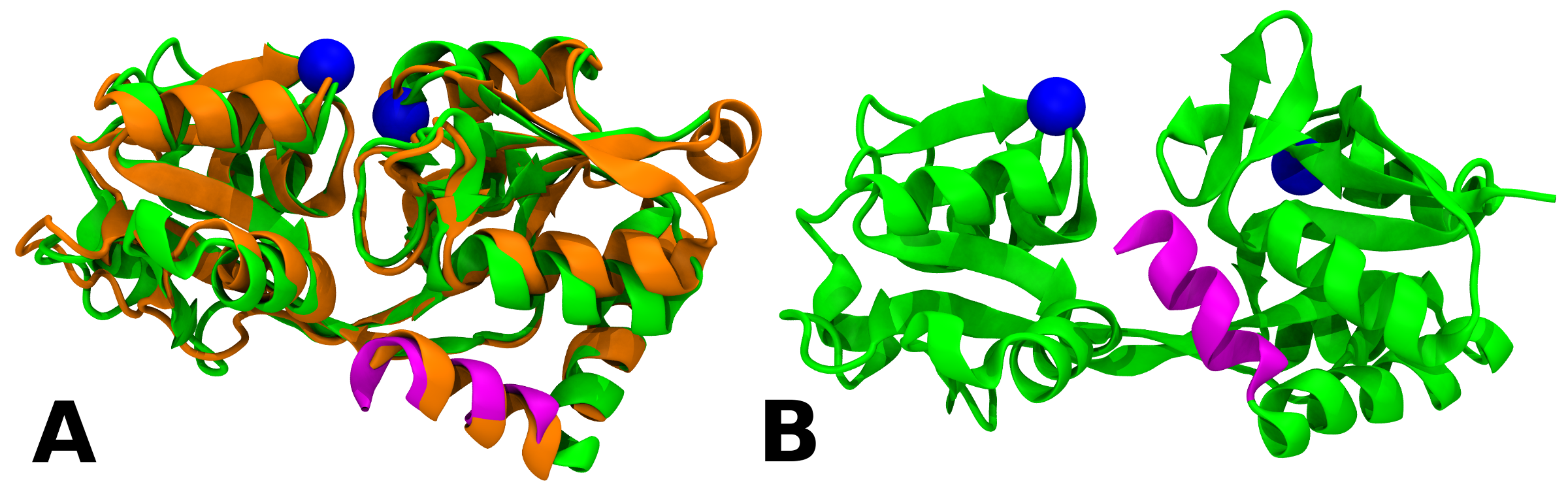
**Figure S2.** **A.** The structure of cs-TmArgBP^CTH^ (orange) aligned with the closed-state conformation of GsArtJ (green) with the CTH colored in magenta. Although the sequence similarity between the two proteins is 30.4, the RMSD between them post structural alignment (in VMD) is ~0.17 nm, which is indicative of high structural similarity between the two. **B.** A snapshot of the open-state conformation of GsArtJ accessed during the MD run. In this open-state conformation one can clearly see the CTH moving between the two lobes at the back of the binding site thereby aiding in the conformational change. The two vdW spheres colored blue indicate the position of Cα-atoms of residues 56-143 in TmArgBP^CTH^ (and identical residues in GsArtJ), which are used to monitor the conformational transition.
